# Exploiting Thin-Film Properties and Guided-Mode Resonance for Designing Ultrahigh-Figure-of-Merit Refractive Index Sensors

**DOI:** 10.3390/s24030960

**Published:** 2024-02-01

**Authors:** Duy Thanh Cu, Hong-Wei Wu, Hung-Pin Chen, Li-Chen Su, Chien-Cheng Kuo

**Affiliations:** 1 Thin Film Technology Center, Department of Optics and Photonics, National Central University, 300, Chung Da Rd., Chung Li, Taoyuan 32001, Taiwan; duythanh@g.ncu.edu.tw (D.T.C.); s215101.ykvs@gmail.com (H.-W.W.); 2 National Applied Research Laboratories, Taiwan Instrument Research Institute, No. 20. R&D Rd. VI, Hsinchu Science Park, Hsinchu 30076, Taiwan; chbin@tiri.narl.org.tw; 3 General Education Center, Ming Chi University of Technology, New Taipei 243303, Taiwan; sulichen@mail.mcut.edu.tw; 4 Organic Electronics Research Center, Ming Chi University of Technology, New Taipei 243303, Taiwan

**Keywords:** refractive index sensor, diffractive grating, high figure of merit, high sensitivity

## Abstract

Guided-mode resonance (GMR) gratings have emerged as a promising sensing technology, with a growing number of applications in diverse fields. This study aimed to identify the optimal design parameters of a simple-to-fabricate and high-performance one-dimensional GMR grating. The structural parameters of the GMR grating were optimized, and a high-refractive-index thin film was simulated on the grating surface, resulting in efficient confinement of the electric field energy within the waveguide. Numerical simulations demonstrated that the optimized GMR grating exhibited remarkable sensitivity (252 nm/RIU) and an extremely narrow full width at half maximum (2 × 10^−4^ nm), resulting in an ultra-high figure of merit (839,666) at an incident angle of 50°. This performance is several orders of magnitude higher than that of conventional GMR sensors. To broaden the scope of the study and to make it more relevant to practical applications, simulations were also conducted at incident angles of 60° and 70°. This holistic approach sought to develop a comprehensive understanding of the performance of the GMR-based sensor under diverse operational conditions.

## 1. Introduction

Guided-mode resonance (GMR) devices have become increasingly popular, with many applications in fields such as sensing, optical communications, and biomedical diagnostics. This unique application is due to the resonant coupling between the incident light and waveguide modes in the grating structure, creating devices capable of highly sensitive and selective detection. Because of this unique feature, GMR devices are made suitable for a variety of applications, including biosensing [1,2,3,4,5], optical filtering [6,7,8,9], and photonic technology [10].

As for refractive index sensors, small changes in the refractive index of the environment are detected by high-precision GMR devices. These devices are increasingly being used in applications such as environmental monitoring, chemical analysis, and label-free detection of biomolecular interactions.

Guided-mode resonance (GMR) is a phenomenon that occurs when diffracted light from a grating is coupled into a waveguide and confined therein. After a certain distance of propagation in the waveguide, the light is coupled out of the grating and interferes with the zeroth-order reflected light, forming a leaky mode. Leaky modes only produce a narrow full width at half maximum (FWHM) under certain phase-matching conditions [11,12]. This phenomenon produces a sharp peak in the reflection spectrum of the GMR device.

GMR gratings exhibit narrow line width, high diffraction efficiency, and sensitivity to the ambient refractive index (RI). GMR biosensors are particularly promising due to their high sensitivity and ultra-narrow linewidth. When the ambient RI changes, the GMR grating produces a corresponding shift in wavelength, which can be used to detect even the smallest changes in RI.

The performance of an RI sensor is evaluated by two key parameters: sensitivity (S) and figure of merit (FOM). S is defined as the change in the wavelength shift divided by the change in the RI; S = Δλ/Δn (nm/RIU) [13]. It plays a vital role in evaluating the sensing capability. The grating structure of the sensor can be modified to tune its sensitivity [14,15,16]. There have been several effective grating structure-related approaches utilized to enhance both S and FWHM, such as modifying the material substrate [17] and thickness of the waveguide layer [18] or expanding the subsurface cavity [19].

Another important factor that determines the performance of an optical resonant sensor is FOM, which is defined as the ratio of the S to the FWHM; FOM = S/FWHM (1/RIU). The FOM is a comprehensive indicator of sensor performance [20,21,22,23], and a sensor with a high FOM will have superior detection capabilities and lower detection limits. Recently, there has been a growing interest in developing new grating structures to improve the FOM of GMR sensors. Some promising approaches include single-layer gratings [24], low-RI substrate grating [25], dislocated double-layered metal gratings [26], and composite constructions [27,28]. However, such sensors are challenging to manufacture and are prone to damage.

The potential to generate highly sensitive surface plasmon resonance (SPR) or local SPR has also attracted significant interest in improving the FOM. However, the use of metal components in SPR leads to the occurrence of ohmic loss, hindering the ability to shrink the FWHM and subsequently resulting in a comparatively low FOM [2,7,29]. As a solution, the adoption of non-metallic materials [1,25,27,28,30] can circumvent the issue of ohmic losses and obtain narrow FWHM. Table 1 displays various sensor designs based on GMR, organized in ascending order of FOM, ranging from smaller to larger values.

G. Lan et al. [1] developed an all-dielectric GMR-based RI sensor, which consists of a simple one-dimensional (1D) grating nanostructure. This device was able to narrow the GMR linewidth to 2 × 10^−2^ nm, resulting in an FOM value of up to 12,000. In this design, the distribution of electric field energy predominantly extended beyond the grating region, leading to a wider FWHM. To achieve an optimal FWHM, it is crucial to confine the electric field within the waveguide region. By accomplishing this confinement, the amount of light leakage can be reduced, resulting in a narrower FWHM. The enhancement of effective medium theory offers a promising approach to redirect the energy distribution from being confined primarily within the grating to an equivalent waveguide layer. This redistribution of energy allows for more efficient confinement within the waveguide, which can lead to improved control over the FWHM. By optimizing the effective medium properties, the design can effectively steer the electric field energy toward the desired waveguide region, promoting a narrower FWHM and enhancing the performance of the sensor. By using a thin film layer to enhance the equivalent RI and confine the electric field within the waveguide, the design achieves better control over the FWHM, leading to improved sensing capabilities. Zhou et al. proposed a design for a structure built from gratings with a thin film deposited on the surface [25]. Their experimental results demonstrated an FOM value up to 4200 ± 600, which is 48 times larger than typical GMR sensors, and this was accompanied by a narrow linewidth (56 pm), high S (233.35 nm/RIU), and a low detection limit of 1.93 × 10^−6^.

This study proposes a simulation of a simple-structured one-dimensional (1D) GMR grating with a thin film layer to enhance the FOM. The electric field intensity within the grating was thoroughly analyzed to assess the improvement in FOM. By modifying various geometric parameters such as the incident angle, grating height, filling factor, and thickness of the film layer on the grating, a high-performance RI sensor operating in the near-infrared spectral range was achieved. The simulated GMR grating structure demonstrated exceptional performance, boasting a high S of 252 and an ultrahigh FOM of 839,666, coupled with an impressively narrow FWHM of 2 × 10^−4^ nm. In addition to the concentrated exploration of specific angles of incidence, simulations for a variety of other angles (60°, 70°) were also undertaken. This expanded perspective aimed to transcend the limitations of a narrow parameter range, broadening the applicability across diverse practical scenarios. This simple-to-fabricate GMR-based all-dielectric sensing device holds great promise for realizing high-performance and compact broadband RI sensors in biochips, electronic tongues, and electronic noses, among other applications.

## 2. Results and Discussion

**Figure 1:**
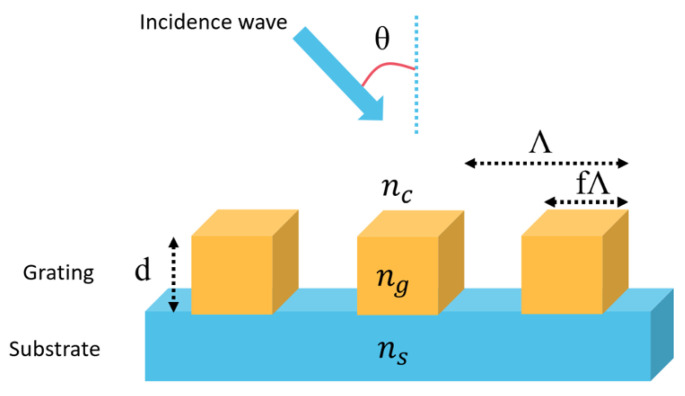
Schematic diagram of the GMR grating, consisting of a dielectric material (n_g_ = 1.6) periodically arranged on a glass substrate (n_s_ = 1.5) with grating height (d = 400 nm), period (Λ = 300 nm), and filling factor (f = 0.9). The system was placed in a medium of refractive index (n_c_ = 1.4) and incident angle (θ = 60°).

The schematic diagram of the proposed GMR grating is shown in Figure 1. The used grating and substrate had refractive indices of n_g_ = 1.6 and n_s_ = 1.5, respectively. The nanostructure they investigated was specifically designed to support guided-mode resonance (GMR) under TM polarization.

As previously mentioned, GMR causes a unique peak in the reflectance spectrum. The eigenvalue equation for the TM field within the waveguide is used in Equation (1) [1,40].

(1) $$\tan(k_{o}d)=\frac{n_{av}^{2}k\left(n_{s}^{2}\gamma +n_{c}^{2}\delta \right)}{n_{c}^{2}n_{s}^{2}k^{2}-n_{av}^{4}\gamma \delta }$$

Here, the wave vector is denoted by $k_{o}$, and the average refractive index is denoted by $n_{av}$. The notations are used in Equation (1), which can be expressed as follows:

(2) $$k_{o}=\frac{2\pi }{\lambda }$$

(3) $$n_{av}=\frac{n_{g}n_{c}}{\sqrt{fn_{c}^{2}+(1-f)n_{g}^{2}}}$$

(4) $$\beta_{i}=k_{o}(n_{c}\sin\theta -i\frac{\lambda }{\Lambda })$$

(5) $$k=\sqrt{{n_{av}}^{2}k_{o}^{2}-\beta_{i}^{2}}$$

(6) $$\gamma =\sqrt{\beta_{i}^{2}-n_{c}^{2}k_{o}^{2}}$$

(7) $$\delta =\sqrt{\beta_{i}^{2}-n_{s}^{2}k_{o}^{2}}$$

$\beta_{i}$ in the preceding formulations is related to the angle of incidence and must meet the following inequality:

(8) $$m\mathrm{ax}\left\{n_{c},n_{s}\right\}\leq \left|\beta_{i}/k_{o}\right|<n_{av}$$

The eigenvalue equation of GMR (1) should be solved with integers i equal to 1 to determine the location of the GMR peak wavelength.

The grating structure was simulated with the Rsoft Photonics CAD Suite Version 2020.09 software, which is based on the rigorous coupled-wave analysis method [41] to visualize the optical response of the sensor device. For the simulation of the GMR sensor, a periodic boundary condition was applied to accurately capture the optical behavior of the grating structure. The simulation domain was defined to represent a unit cell of the periodic grating, ensuring that the optical effects within the cell could be extrapolated to the entire structure. Additionally, the optical properties of the materials, such as refractive indices and absorption coefficients, were carefully defined to ensure realistic representation of the physical system. Initially, the diffraction efficiency of the nanostructure was simulated under TM-polarized light with an incident angle θ = 60°, grating height d = 400 nm, period Λ = 300 nm, and filling factor f = 0.9. The used grating and substrate had refractive indices of n_g_ = 1.6 and n_s_ = 1.5, respectively.

**Figure 2:**
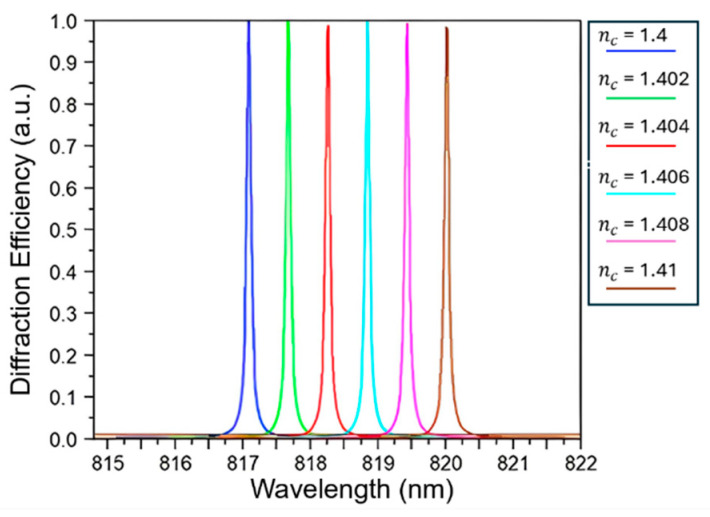
Effects of varying environmental refractive indices ranging from 1.4 to 1.41 with each step of 0.002 on the resonance peak position.

In accordance with inequality (2), the detection range of this sensor is confined to ambient RI between 1.15 and 1.55. As depicted in Figure 2, a simulation was conducted in which the RI of the surrounding medium was varied within a narrow range to demonstrate its sensing capability, specifically from 1.4 to 1.41 with each step of 0.002. The S of the sensor was calculated based on the change in peak wavelength (Δλ) from 817.089 nm to 820.015 nm, which corresponds to a change in the refractive index (Δn) of 0.01. This calculation yielded an S value of 292.6 nm/RIU and an FWHM of 7.3 × 10^−2^ nm, resulting in an FOM of 4008.2 (1/RIU).

In the next section, we demonstrate that modifying the geometry of the nanostructure and overlaying a thin film layer on the grating reduced the FWHM, improved both the S and FOM of the sensor, and enabled the detection of smaller changes in RI, resulting in better overall performance. The enhancement of the FOM is a critical aspect, as it signifies the sensor’s ability to distinguish minute changes in the RI, thereby improving the overall performance of the sensor. This discussion will further delve into the implications of these modifications on the sensor’s performance and potential applications.

### 2.1. Incident Angle

**Figure 3:**
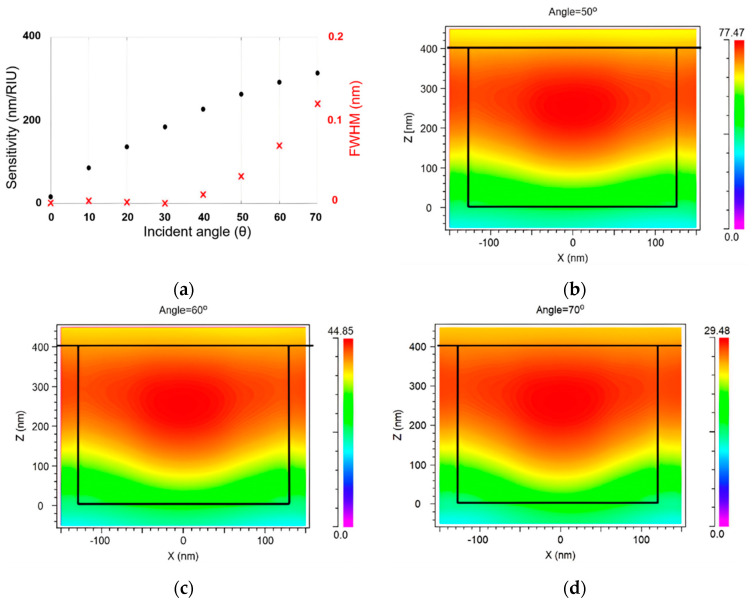
(**a**) The correlation between S, FWHM, and incident angle in the range of 0° to 70°; TM electric field intensity at different angles: (**b**) θ = 50°, (**c**) θ = 60°, (**d**) θ = 70°.

Numerous studies have demonstrated that GMR is highly sensitive to incident angles, even at relatively low ones [1]. Our model was conducted with a variable incident angle to investigate the relationship between the incident angle and GMR, while keeping the other grating parameters constant. Simulations were performed over a range of incident angles from 0° to 70°. The relationship between S and FWHM as a function of incident angle is shown in Figure 3a. As the angle of incidence increased, the effective optical path length of light in the grating structure increased due to its geometry. This enhanced the coupling of light into the grating’s guided modes, increasing the shift in the resonance wavelength for a given change in refractive index, resulting in a more sensitive sensor. However, the resonance peak also broadened, increasing the FWHM because of the increased interaction of light with the grating at higher incident angles.

At small incident angles ranging from 0° to 40°, the FWHM was exceptionally narrow, but the S was relatively low, limiting the sensor’s capabilities. Therefore, while designing the grating for the sensor, an appropriate angle of incidence must be determined to strike a compromise between the S and FWHM. It was found that an incident angle range of 50°–70° was suitable for achieving high S while maintaining the narrow FWHM. In this range, the sensitivities were 269, 293, and 336, with an incidence angle of 50, 60, and 70, respectively. The FWHM exhibited a remarkably narrow value of 3.9 × 10^−2^ nm at a 50° incidence angle, progressively increasing to 1.38 × 10^−1^ nm at 70°. As a result, the FOM dropped from 6878.8 (1/RIU) to 2433 (1/RIU) when the incident angle was increased from 50° to 70°.

By examining data across a range of angles from 50° to 70°, a clear correlation emerged between the angle of incidence and the electric field intensity (EFI), as shown in Figure 3b–d. This visually demonstrates that, as the angle of incidence increased, the electric intensity diminished. The maximum EFI decreased from 77.47 Newton/Coulomb (NC^−1^) to 29.48 NC^−1^ as the angle of incidence increased. This phenomenon can be attributed to the way the electric field energy is distributed within the grating when the angle of incidence is small. Under these conditions, most of the electric field energy is confined within the grating, resulting in reduced leakage of light. This makes the spectral line associated with the resonance peak sharper and narrower, which results in a narrower FWHM. This characteristic is desirable in RI sensors, as it enhances their precision. This highlights the importance of controlling the incidence angle to achieve the desired electric field distribution and intensity.

Our preliminary findings indicated that the incident angle has a significant impact on the performance of GMR-based sensors, including S, FWHM, and FOM. However, these relationships are not purely linear and, thus, necessitate a comprehensive study to achieve an optimal balance. To this end, we embarked on a two-pronged approach. Initially, we selected a specific incident angle, holding it constant while varying other grating parameters to explore their impacts on the GMR. By isolating the effect of the incident angle, we hoped to establish a deeper understanding of the phenomena at play and reveal key correlations between the grating parameters and the sensor’s performance. Subsequently, we extended our analysis to three distinct incident angles, each representing different practical applications. By exploring the sensor behavior at these angles, we aimed to derive more universally applicable insights into the design of GMR-based sensors. In our investigation, the focus was on generating a sensor with a high FOM. The preliminary investigations suggested an incident angle of 50° as a promising starting point for further exploration. This choice was driven by the desire to achieve a narrow FWHM that satisfies the desired criteria.

### 2.2. Grating Height

**Figure 4:**
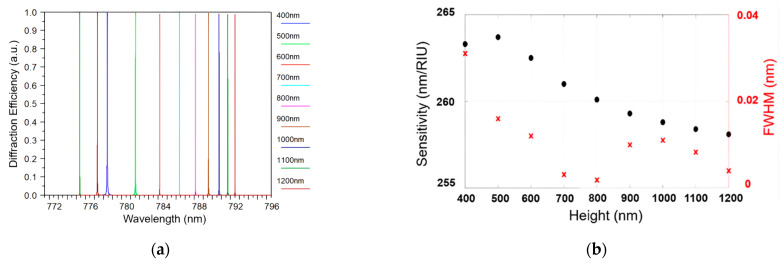
(**a**) Diffraction efficiency as a function of grating height in the range of 400 nm to 1200 nm; (**b**) correlation between S, FWHM, and grating height.

The investigation into the impact of grating height on the FWHM was undertaken to uncover the relationship between these two parameters and the overall performance of the sensor. To conduct this evaluation, the grating height was systematically varied, while maintaining a constant grating period and filling factor, all under an incident angle of 50°. Specifically, the grating height was increased from 400 nm to 1200 nm, and the simulation results illustrated a corresponding shift in the position of the resonance peak from 777.5 nm to 791.5 nm, as depicted in Figure 4a.

Our simulations revealed a significant decrease in the width of the resonant peak as the grating height was increased from 400 nm to 1200 nm. This decrease in the width of the resonant peak holds crucial importance as it directly influenced the overall performance of the sensor, as is further discussed later in the study.

Figure 4b clearly shows how the grating height affected the S and FWHM of the resonance peak. It illustrates that, as the grating height increased from 400 nm to 800 nm, the FWHM decreased from 3.1 × 10^−2^ nm to 1.85× 10^−3^ nm. However, beyond the grating height of 800 nm, the FWHM started to increase once again. This phenomenon can be explained by the interaction between the bound mode and radiation mode within the waveguide grating. When these modes were orthogonal, the coupling loss between them was minimized. This reduction in coupling loss led to a narrower FWHM of the resonance peak. Additionally, the narrowing of the FWHM resulted in a significant increase in the FOM of the sensor. In the analyzed data, the FOM exhibited a remarkable improvement, increasing from 8493 (1/RIU) to 140,594 (1/RIU) when the grating height was optimized.

**Figure 5:**
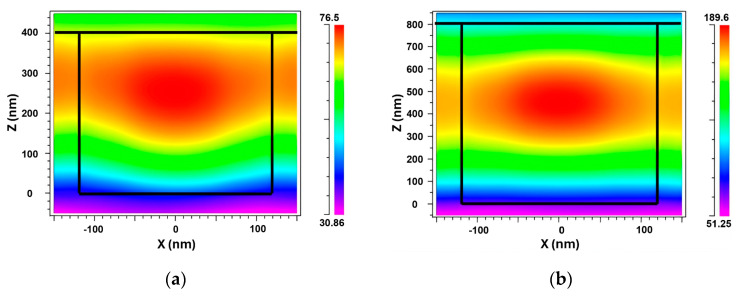
TM electric field intensity at grating heights of (**a**) 400 nm and (**b**) 800 nm.

Figure 5 shows how the electric field was distributed in the grating for two different grating heights: 400 nm and 800 nm. Our results revealed an increase in the EFI as a result of the grating height modification. Specifically, the maximum EFI rose from 76.5 NC^−1^ to 189.6 NC^−1^, signifying a substantial enhancement. The plots unmistakably illustrate that the electric field intensity was amplified when the grating height was optimally set to 800 nm. This outcome can be attributed to the reduced coupling loss achieved at this particular height, enabling a more confined propagation of light within the grating. Consequently, this confinement contributed to an improvement in the FOM, a critical metric for sensor performance evaluation.

Based on these findings, we reached the conclusion that a grating height of 800 nm is the optimal choice for future simulations. By employing this specific height, the sensor will operate at its peak performance level, exhibiting a heightened S and a narrower FWHM. These characteristics are vital for the realization of high-performance sensors that deliver precise and accurate measurements.

### 2.3. Filling Factor

**Figure 6:**
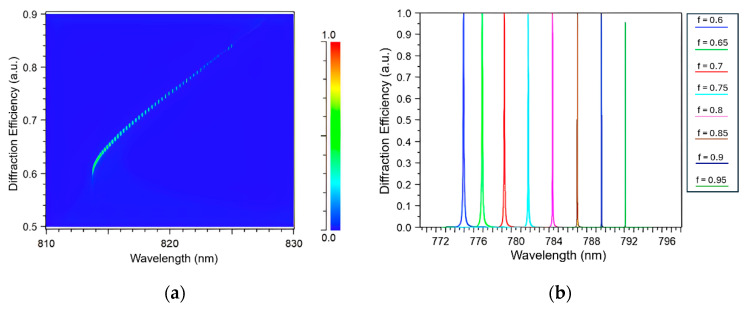
(**a**) Shift of the resonance position with the filling factor (0~1). (**b**) Diffraction efficiency as a function of the filling factor (0.6~0.95).

It is well-known that the position of the resonance peak can be significantly influenced by various structural parameters. In this section, the impact of the filling factor on the resonance position of the grating was also investigated. Therefore, it was important to evaluate the effect of the filling factor on the resonance position in this context. Figure 6a shows the shift of the resonance peak as the filling factor increased from 0 to 1. It can be observed that the resonance point experienced a shift in position as the filling factor was varied. However, it is worth noting that the resonance point was eventually cut off at a certain wavelength due to the influence of the Rayleigh threshold wavelength [1], which satisfies the condition described by Formula (9):

(9) $$\lambda =\Lambda (n_{s}+n_{c}\sin\theta )$$

Therefore, to explore the impact of the filling factor on the grating’s performance, simulations were conducted within a range of 0.6 to 0.95. Figure 6b depicts the variation in the diffraction efficiency as the filling factor increased, assuming a grating period of 300 nm, a grating height of 800 nm, and an incident angle of 50°. The simulation results demonstrate that as the filling factor increased, the resonance peak of the grating shifted towards longer wavelengths. This phenomenon can be attributed to the principles of effective environment theory and waveguide theory, as described in a previous study [42]. Initially, based on the principle of equivalent RI, the formulas used to describe the behavior of the grating can be expressed as follows:

(10) $$n_{TE}=\sqrt{(1-f)n_{g}^{2}+fn_{c}^{2}}$$

(11) $$n_{TM}=\sqrt{\frac{n_{g}^{2}n_{c}^{2}}{fn_{c}^{2}+(1-f)n_{g}^{2}}}$$

According to Formulas (10) and (11), as the filling factor increases, the equivalent RI of the grating layer also increases. Subsequently, when these values are substituted back into Formula (1), the resonance peak of the grating shifts due to the change in the equivalent RI. Figure 6b also shows that the peak width gets narrower as the filling factor rises, which can be explained by the light electric field distribution in the grating. It is noteworthy that a filling factor of 0.95 yielded the smallest FWHM according to the observations in Figure 6b. However, for the original grating construction, a filling factor of 0.9 was utilized instead of higher values. This choice was made because, when the filling factor exceeds 0.9, there may not be sufficient space available to accommodate the deposition of a thin film on top of the grating. Therefore, to ensure the feasibility of incorporating a thin film layer, a filling factor of 0.9 was selected for the subsequent simulations.

**Figure 7:**
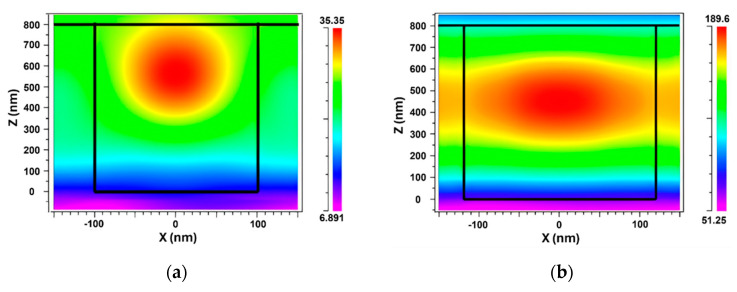
TM electric field intensity at different filling factors of (**a**) f = 0.6, (**b**) f = 0.9.

Figure 7 provides a visual comparison of the electric field distribution diagrams for two different filling factors: 0.6 and 0.9. It is evident from the diagrams that as the filling factor increased from 0.6 to 0.9, there was a substantial increase in the EFI from 35.35 NC^−1^ to 189.6 NC^−1^. This increase in the EFI can be attributed to the corresponding increase in the equivalent RI of the grating structure. As the filling factor increased, the equivalent RI rose, resulting in a more efficient confinement of the light within the structure. By achieving a higher EFI through an increased filling factor, the FOM was enhanced, indicating improved performance and capabilities of the system.

### 2.4. Simulation of Multiple Parameters for Optimal Grating

**Figure 8:**
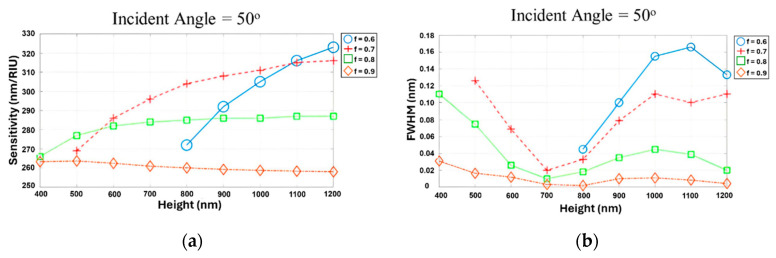
(**a**,**b**) Simultaneous simulation of S and FWHM as functions of the grating height (400~1200 nm) and filling factor (0.6, 0.7, 0.8, 0.9) at 50° incidence.

The investigation of individual parameters was conducted to understand their impact on the performance of the RI sensor. It was essential to verify the results obtained from investigating individual grating design parameters by conducting simulations that consider all parameters simultaneously. Figure 8a,b presents the simulation results for the S and FWHM at an incidence angle of 50°, considering variations in the grating height and filling factor. Based on the simulation results, the optimal structure was identified, consisting of an incident angle of 50°, a grating height of 800 nm, and a filling factor of 0.9. This demonstrates that the optimal parameter results for the grating were invariant under simultaneous and sequential parameter simulations, providing robust validation of the design approach. These parameters were found to yield desirable performance characteristics for the RI sensor. Using this optimal configuration, the simulation predicted that the FWHM can be reduced to 0.00185, indicating a significantly narrower spectral linewidth. Additionally, the S was estimated to be approximately 260, suggesting a high level of responsiveness to changes in the refractive index. The calculated FOM value for this optimal structure was 144,500, signifying a strong FOM. Subsequently, a thin film layer was applied onto this optimized structure to further investigate its impact on the sensor’s performance. The investigation of the thin film layer is discussed in the next section, providing additional insights into the enhancement of the RI sensor.

### 2.5. Effect of the Thin Film

The impact of the incident angle, grating height, and filling factor on the S and FOM in resonance-based sensors was comprehensively investigated and simulated in the earlier sections of this study. The primary objective was to confine the electric field energy within the grating structure, aiming for a narrow FWHM for improved sensor performance. To achieve this, the structural design incorporated a higher equivalent RI, effectively trapping the energy in the equivalent waveguide layer rather than dispersing it within the grating. This strategic design choice resulted in reduced light leakage, narrower FWHM, and enhanced FOM.

**Figure 9:**
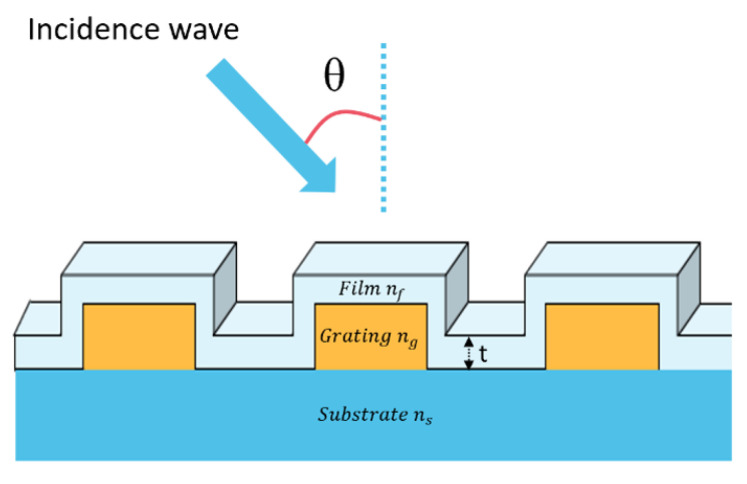
Schematic of the optimal GMR grating consisting of a dielectric material (n_g_ = 1.6) periodically arranged on a glass substrate (n_s_ = 1.5) with a grating height of 800 nm, period of 300 nm, and filling factor of 0.9, coated with a thin film of refractive index n_f_ and thickness t. The system was placed in a medium with a refractive index of 1.4 and an incident angle of 50°.

In this section, the impact of a film layer on the FOM was investigated using the previously optimized grating parameters. A uniform film layer of consistent thickness was applied to the top and side faces of the grating structure, as depicted in Figure 9. The structural characteristics considered for this analysis included a grating period of 300 nm, an incident angle of 50°, a filling factor of 0.9, and a grating height of 800 nm. This study examined the changes in diffraction efficiency as the RI of the coating layer increased from 1.4 to 2.4, accompanied by a corresponding increase in the thickness of the film layer from 0 nm to 10 nm. These variations in the film characteristics were systematically analyzed to understand their influence on the overall performance of the structure and the resulting FOM.

**Figure 10:**
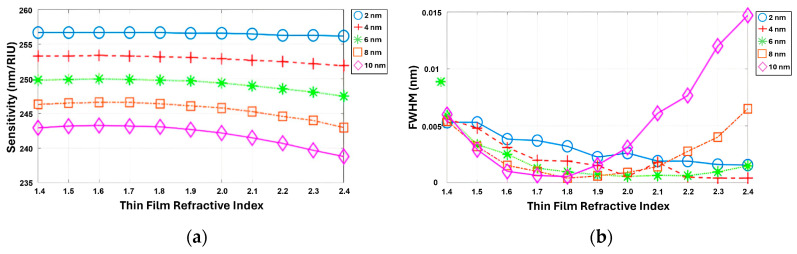
(**a**,**b**) Simultaneous simulation of S and FWHM depends on the refractive index of a thin film varying from 1.4 to 2.4, with thicknesses of 2, 4, 6, 8, and 10 nm.

It was observed that the S decreased as the film thickness increased, while the FWHM reached its maximum value at a specific refractive index and film thickness, as shown in Figure 10a,b. Here, two phenomena occurred, i.e., the enhancement of the equivalent RI and the orthogonality of the bond mode and radiation mode, leading to the FWHM narrowing. Specifically, when the film covers the grating surface, it indirectly raises the grating height, which will increase the equivalent RI and coupling loss [43]. However, when the equivalent waveguide thickness and index reach a certain value, the bond and radiation modes become orthogonal, causing the coupling loss to sharply drop [43]. As a result, the FWHM gradually decreased until it reached its minimum value at a film thickness of 4 nm. Beyond this point, the FWHM started to increase as the film thickness continued to increase.

**Figure 11:**
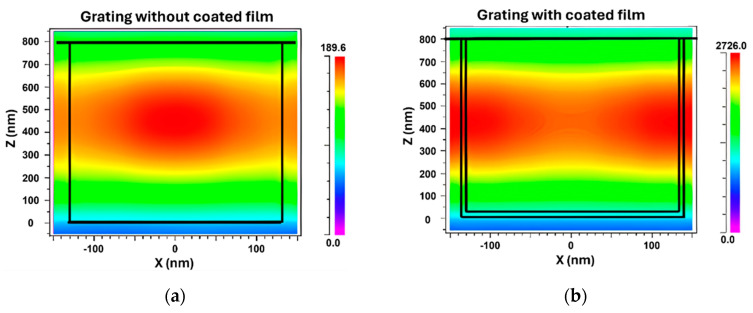
TM electric field distribution of grating (**a**) without and (**b**) with coated films.

To evaluate the impact of a coated film on the electric field distribution and FWHM of the grating, a simulation was conducted comparing gratings without and with a film. The parameters that yielded the narrowest FWHM from Figure 10b were selected as the starting point to investigate the differences in electric field distribution. Figure 11 illustrates the TM electric field distribution of the grating without and with coated films. The influence of the film coating on the FWHM of the grating was evident. In the absence of a film, the electric field strength reached a maximum value of approximately 189.6. However, when a thin film was present on the grating surface, the intensity of the dispersed electric field was significantly increased, with a value of 2726 observed for a refractive index of 2.4 and a film thickness of 4 nm.

When a film is coated on the grating, the equivalent refractive index and waveguide thickness increase, leading to a change in the light distribution. As a result, a significant portion of the light becomes concentrated within the film layer, which effectively functions as an equivalent waveguide [44]. This concentration of light within the waveguide layer is attributed to the higher equivalent refractive index and increased waveguide thickness caused by the presence of the film. From the analysis of the simulation data, it was evident that the most effective configuration for an RI sensor is one that achieves an S of 252, an FWHM of 2 × 10^−4^ nm, and an FOM of 839,666. Table 2 illustrates a significant enhancement of the FOM for gratings with a coated film compared to non-coated gratings. The FOM value of a coated grating was obtained to be nearly six times that of an uncoated grating.

In experimental investigations, numerous materials, including Zirconium dioxide (ZrO_2_), Tantalum oxide (Ta_2_O_5_), and Titanium dioxide (TiO_2_), etc., each possessing a refractive index of approximately 2.4 nm, could be considered suitable candidates for deposition onto grating structures through the process of atomic layer deposition. These results underscore the importance of coating a film on the grating structure, as it leads to an increase in the equivalent refractive index and waveguide thickness, resulting in a concentration of light within the film layer. This optimized configuration contributes to the improved performance of the RI sensor, as indicated by the high S and FOM values obtained.

### 2.6. Expansion for Distinct Incident Angles

Adopting the simulation methodology previously applied to the 50° incident angle, we then conducted similar analyses for incident angles of 60° and 70°. This step represented a significant expansion in our analysis, with the potential to offer a more complete picture of the GMR sensor’s response to variations in incident angles. By systematically varying the incident angle, we aimed to identify trends and behaviors that were not immediately evident at a single angle of incidence.

**Figure 12:**
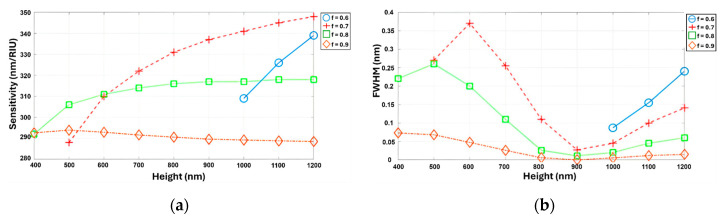

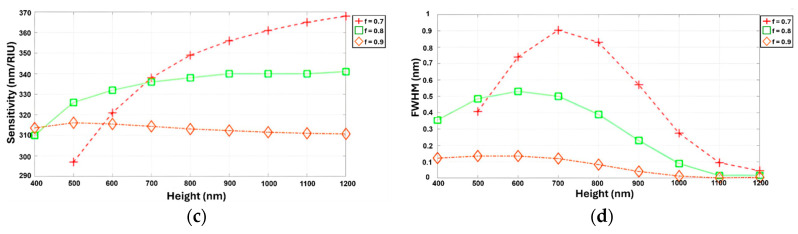
Simultaneous simulation of S and FWHM, with a filling factor between 0.6 and 0.9, for grating heights between 400 and 1200, corresponding to different incident angles: (**a**,**b**) 60°; (**c**,**d**) 70°.

Upon identifying the optimal parameters in the preceding chapters, we then shifted our focus to the influence of the film coating on the grating structure. We selected the structural parameters that yielded the narrowest FWHM, as presented in Figure 12. The selected parameters are shown as follows in Table 3. We then simulated the application of a high refractive index film onto this optimized grating structure to investigate its effects on both S and FOM.

In the subsequent simulations, the refractive index of the film coating on the structure varied from 1.4 to 2.4. In parallel, we examined the changes in S and FWHM as the thickness of the film increased incrementally from 0 nm to 10 nm.

**Figure 13:**
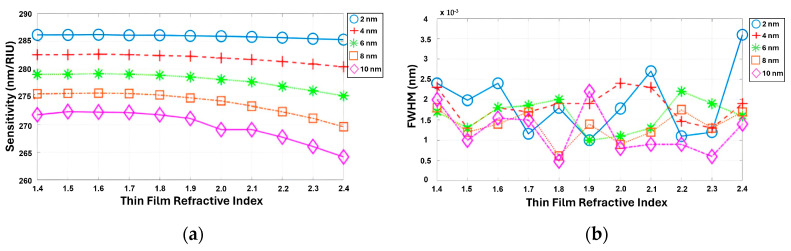

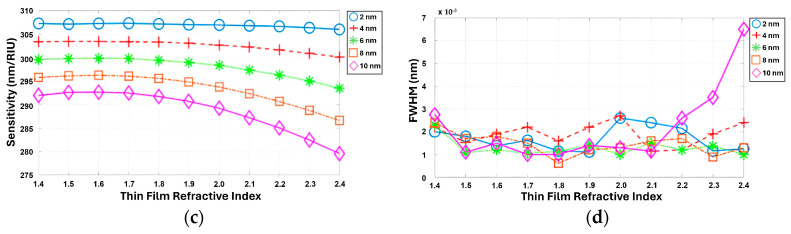
The simultaneous simulation of S and FWHM depends on the refractive index of a thin film varying from 1.4 to 2.4, with thicknesses of 2, 4, 6, 8, and 10 nm for different incident angles of (**a**,**b**) 60°and (**c**,**d**) 60°.

From the data presented in Figure 13, it became apparent that we can derive specific parameter sets that yield the most favorable results in terms of both S and FWHM. This deduction is crucial, as it offers insights into the optimal operational conditions for achieving the best sensor performance. To simplify the interpretation and application of these findings, we consolidated the optimal parameter sets for various conditions in Table 4 below. The significant influence of thin film deposition on the grating’s light–matter interaction was further corroborated by observations at varied incident angles (60°, 70°). In both cases, the thin film coating demonstrably narrowed the FWHM of the relevant spectral feature. This narrowing effect can be attributed to the augmented interaction between light and the grating’s periodic structure facilitated by the thin film, ultimately leading to the realization of a highly sensitive sensor with a superior FOM. These organized results not only serve as a valuable guide for designing GMR-based sensors but also facilitate further studies to explore the potential of such sensors under a wider range of conditions.

**Figure 14:**
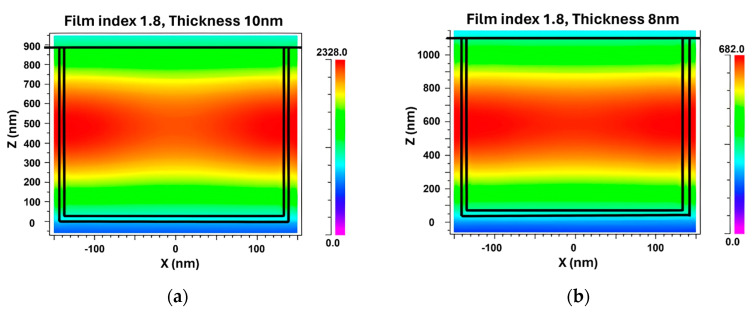
TM electric field distribution of grating parameters at (**a**) 60°and (**b**) 70°.

We noticed a substantial amplification in electric field intensity following the application of a film layer to the grating, as shown in Figure 14. The maximum EFI value increased gradually from 682 NC^−1^, 2328 NC^−1^, and 2726 NC^−1^, corresponding to the decreasing angle of incidence of 70°, 60°, and 50°, respectively. During this state, energy confinement within the grating was enhanced, inducing a contraction of the FWHM. This aligns with our initial hypothesis regarding the influence of incident angle and grating structure on the electric field distribution. This deeper comprehension of the phenomenon is paramount in fine-tuning the GMR-based sensor’s performance, especially in terms of its spectral resolution, signified by the FWHM.

## 3. Conclusions

We proposed and optimized a label-free RI sensor based on a waveguide grating structure. An in-depth analysis and explanation of the relationship between the grating parameters, including the angle of incidence, height, filling factor, and S and FWHM were carried out. To further enhance the performance of the sensor, a thin film was coated onto the grating surface. This simultaneously increased the equivalent refractive index and confined the electric field within the waveguide, significantly reducing light leakage. By simulating gratings at 50°, we identified an optimal design with a refractive index of 2.4 nm and a film thickness of 4 nm. This configuration achieved a remarkable sensitivity of 252 nm/RIU while maintaining an incredibly narrow full width at half maximum of 0.0002 nm. These outstanding characteristics translate to an exceptional figure of merit (FOM) of 839,666. The proposed grating-based refractive index sensor demonstrated a clear trade-off between S and FWHM with increasing incident angle. At higher incident angles of 60° and 70°, the sensor sensitivities increased to 272 nm/RIU and 296 nm/RIU, resulting in figures of merit (FOM) of 617,700 and 341,745, respectively. These highly sensitive sensors and their high FOM values are critical to accurately detecting and quantifying even the slightest changes in biological systems, environmental and chemical changes, and even in food.

## Figures and Tables

**Table 1:** Previously existing sensor designs based on GMR.

Reference	Modalities	S (nm/RIU)	FOM (1/RIU)
[31]	Silver-shell nanorods with air cores	1200	26.67
[24]	1D grating with deep grating	229.43	31.52
[32]	PR grating coated Au thin film	553	38
[33]	Al_2_O_3_ gratings/Si/Au/Si	440.295	50.30
[30]	Si_3_N_4_ grating/glass	375	68
[34]	Au nanocubes/SiO_2_/Au	1002.0	417.0
[35]	1D metal grating in dielectric cavity	800	1337
[36]	HfO_2_ grating/Au/SiO_2_	550	1571.4
[37]	Si_3_N_4_ gratings/SiO_2_	266	1662.5
[25]	UV curable resin coated TiO_2_	233.35	4200 ± 600
[38]	TiO_2_ grating/glass	657	9112
[1]	All-dielectric nano-silt array	300	12,000
[27]	Sidewall gratings in dual-slot waveguide	661	12,018
[16]	Different dielectric-based GMR	1427.3	16,457
[39]	1D grating with polymer	63.9	21,300
[28]	Stacked resonant compound gratings	401.8	57,404

**Table 2:** S and FWHM correspond to grating (a) without and (b) with coated films.

Grating	S (nm/RIU)	FWHM (nm)	FOM (1/RIU)
Non-coating	260	1.85 × 10^−3^	144,500
Coating	252	2 × 10^−4^	839,666

**Table 3:** Optimal parameters for simultaneously simulating diffraction gratings at 60° and 70° incident angles, along with their corresponding S and FOM.

Incident Angle	Period (nm)	Filling Factor	Height (nm)	S	FOM (1/RIU)
60°	300	0.9	900	289.6	231,680
70°	300	0.9	1100	311	296,190

**Table 4:** The optimal parameter sets at incident angles of 60°and 70°.

Incident Angle	Refractive Index	Thickness (nm)	S (nm/RIU)	FWHM (nm)	FOM (1/RIU)
60°	1.8	10	271.7	4.4 × 10^−4^	617,700
70°	1.8	8	295.6	8.6 × 10^−4^	341,745

## Data Availability

Data are contained within the article.
